# The Identification of Prohibitin in the Rat Heart Mitochondria in Heart Failure

**DOI:** 10.3390/biomedicines9121793

**Published:** 2021-11-29

**Authors:** Yulia Baburina, Roman Krestinin, Irina Odinokova, Irina Fadeeva, Linda Sotnikova, Olga Krestinina

**Affiliations:** Institute of Theoretical and Experimental Biophysics, Russian Academy of Sciences, 142290 Pushchino, Moscow Region, Russia; byul@rambler.ru (Y.B.); rkrestinin@bk.ru (R.K.); odinokova@rambler.ru (I.O.); aurin@gmail.com (I.F.); linda_sotnikova@mail.ru (L.S.)

**Keywords:** rat heart mitochondria (RHM), heart failure (HF), prohibitin (PHB), astaxantin (AX), isoproterenol (ISO), oxidative stress, oxidative phosphorylation (OXPHOS)

## Abstract

Mitochondria are considered the main organelles in the cell. They play an important role in both normal and abnormal heart function. There is a supramolecular organization between the complexes of the respiratory chain (supercomplexes (SCs)), which are involved in mitochondrial respiration. Prohibitins (PHBs) participate in the regulation of oxidative phosphorylation (OXPHOS) activity and interact with some subunits of the OXPHOS complexes. In this study, we identified a protein whose level was decreased in the mitochondria of the heart in rats with heart failure. This protein was PHB. Isoproterenol (ISO) has been used as a compound to induce heart failure in rats. We observed that astaxanthin (AX) increased the content of PHB in rat heart mitochondria isolated from ISO-injected rats. Since it is known that PHB forms complexes with some mitochondrial proteins and proteins that are part of the complexes of the respiratory chain, the change in the levels of these proteins was investigated under our experimental conditions. We hypothesized that PHB may be a target for the protective action of AX.

## 1. Introduction

Mitochondria play a central role in the life of the cell, providing a balance between pro- and anti-apoptotic factors and providing ATP through the system of oxidative phosphorylation (OXPHOS) [1,2]. The system of OXPHOS is the most efficient pathway to generate energy in cells, and consists of respiratory chain complexes such as NADH-ubiquinone oxidoreductase (Complex I), succinate-ubiquinone oxidoreductase (Complex II), ubiquinone-cytochrome c oxidoreductase (complex III), cytochrome c oxidase (Complex IV), and F_1_F_o_-ATP synthase (Complex V) [1]. Recent experimental data have shown that there is a supramolecular organization between the complexes of the respiratory chain, which is based on specific interactions between individual respiratory complexes [3]. The so-called supercomplexes (SCs) are not only real objects, but are also involved in mitochondrial respiration. The role of SCs correspond to their activity, that is, they provide some advantage in the transfer of electrons [4]. It is suggested that combining the mitochondrial complexes into SCs can have structural or functional benefits, such as preventing their destabilization and degradation, increasing the efficiency of electron transport and substrate channel, and reducing electron or proton leakage [5]. Due to the formation of SCs, there is a structural interdependence between individual OXPHOS complexes [6]. This has serious biological as well as biomedical consequences, since defects in the assembly of mitochondrial complex enzymes can cause severe encephalomyopathies and neurodegenerative disorders in humans and are associated with the aging process [7,8].

There is evidence that prohibitin proteins (PHBs) are involved in the regulation of OXPHOS activity by interacting with some subunits of the OXPHOS complexes, modulating their stability and translation [9]. It can be emphasized that the role of PHBs is tightly related to the functioning of the complexes of the respiratory chain in mitochondria.

The regulation of mitochondrial function is a complex process involving many mitochondrial proteins. In recent years, the PHBs have received a lot of attention due to their multiple functions in mitochondria. There are two highly homologous subunits in the eukaryotic mitochondrial PHB complex, PHB1 and PHB2, which have 50% amino acid sequence identity and 60% similarity [10]. It has been shown that PHB1 and PHB2 are localized in the nucleus, mitochondria and cytosol, and are associated with specific receptors in cell membranes [11]. In the nucleus, PHBs play the role of co-regulators of transcription, and in the cytosol, PHBs interact with proteins involved in cytoskeletal transport and cell signaling [12]. There is evidence that PHB1 and PHB2 function predominantly in mitochondria [13]. One of the functions of PHBs in mitochondria is to control the quality of mitochondrial protein [14]. The absence of PHB1 leads to instability of the mitochondria-encoded respiratory chain subunits [15]. Moreover, PHBs are involved in the translation of mitochondrial proteins [16].

Heart failure is a multifactorial disease that is a condition with a poor prognosis. Despite the fact that effective methods of treatment have been developed, mortality from heart failure remains high. Therefore, the search for new approaches in the treatment of this disease is urgent [17,18]. There are experimental preclinical studies showing reductions in heart disease through the use of drugs such as coenzyme Q10, resveratrol, lycopene, and a natural inhibitor of leukotrienes involved in mitochondrial damage [19].

Astaxanthin (AX) is a xanthophil ketocarotenoid derived primarily from seaweed (*Haematococcus pluvialis*) and has antioxidant and anti-inflammatory effects [20]. It was noted that the use of AX increased the immune response and reduced the symptoms of cardiovascular diseases and various types of cancer [21,22]. It has been shown that AX can play a key role in suppressing oxidative stress in the myocardial and SH-SY5Y cells [23,24]. We have previously shown that AX increased the activity of respiratory chain complexes under ISO-induced mitochondria dysfunction [25]. We hypothesized that dietary AX supplementation may prevent cardiovascular disease.

In this study, we identified a protein that altered its content in the mitochondria of the heart of rats with heart failure. We hypothesized that this protein could be a target for the protective property of AX.

## 2. Materials and Methods

### 2.1. Animals and Treatment

In the present work, we used Wistar rats (16 animals, weight 240–250 g, age 2 months, males). Four groups of rats were used in the work (four rats in each group). The rats in group 1 were used as control (no additives); AX was used to treat the group 2 rats; the injection isoproterenol (ISO, isoprenaline hydrochloride, Sigma 15627, St. Louis, MO, USA) was used to induce heart failure [26] (the rats in group 3), and rats in group 4 were treated with AX, and then two weeks later injected with ISO (twice).

AX (5%, 150 mg/kg) was administrated as described in [25,27]. ISO was dissolved in physiological saline at a ratio of 100 mg per kg of rat weight and administered to the animals two times with an interval of 24 h to induce mitochondrial disturbances. The experiments were carried out according to the Regulations for Studies with Experimental Animals (Decree of the Russian Ministry of Health of 12 August 1997, No. 755). The protocol was approved by the Commission on Biological Safety and Ethics at the Institute of Theoretical and Experimental Biophysics, Russian Academy of Sciences (February 2021, protocol N18/2021).

### 2.2. Isolation of Rat Heart Mitochondria

Mitochondria were isolated from the whole heart according to the method described in [28]. The heart was purified from blood vessels, crushed and homogenized. The homogenate was suspended in the isolated medium containing 75 mM sucrose (Sigma S7903, Saint-Louis, MO, USA), 10 mM Tris-HCl (pH 7.4), 225 mM mannitol (Sigma M4125, Saint-Louis, MO, USA), 0.5 mM EDTA (Sigma E9884, Saint-Louis, MO, USA), 0.5 mM EGTA (Sigma E3889, Saint-Louis, MO, USA), and 0.1% BSA (Sigma A6003, Saint-Louis, MO, USA). Mitochondria were isolated by differential centrifugation. At first, the homogenate was centrifuged at 1000 × *g* for 10 min to precipitate blood and destroyed mitochondria. The supernatant was precipitated at 8500 × g for 10 min. The precipitated RHM were washed and in isolation medium without EDTA or BSA (8500× *g*, 10 min). All procedures were conducted at 4 °C. The concentration of protein RHM was determined using the Bradford assay (30–35 mg/mL).

### 2.3. Histological Analysis

Identical areas of the left ventricle (LV) of the rat heart (upper right quadrant, above the left coronary artery) were taken to analyze fibrotic lesions of the heart. The LV fragments were dissected from the whole heart with a scalpel immediately after extraction from the rat thorax and rapidly washed from blood in cold phosphate-buffered saline. LV was fixed in neutral buffered formalin at room temperature for 24 h according to the procedure described in [29]. Then, the LV fragments were washed in distilled water and placed with Compound Tissue Tek (Sakura, Tokio, Japan) at 4 °C for 12 h. Each three consecutive 9 μm thick LV transverse sections were obtained using a cryotome (Shandon CRYOTOME 620E, Thermo Fisher Sci., Waltham, MA, USA) with a step of 30 μm. The obtained sections were stained with hematoxylin and eosin (H&E), as well as two differential trichromic stains. Histotopograms were obtained on a Nikon Eclipse Ti-E microscope (Nikon, Tokio, Japan) and using the Nis Elements AR4.13.05 software (Build 933). General myocardial fibrosis was assessed by two methods: Masson’s trichrome staining and Lilly’s trichrome staining [30]. The percentage of fibrotic alterations in the LV tissues from each group of rats was assessed by digitized images (five areas of analysis from each area) using the non-commercial program ImageJ (https://imagej.nih.gov/ij/ (accessed on 16 September 2021)). The maximum fibrosis observed in the section (blue areas) was calculated as the area taken by connective tissue and cardiac myocytes ×100, as described in [31].

### 2.4. The Preparation of Samples, Electrophoresis, and Immunoblotting of Mitochondrial Proteins

The preparation of tissue lysate, ~6–7 mg of heart tissue from left ventricular was taken. After adding ice-cold buffer (RIPA buffer and protease cocktail) to a piece of heart tissue, the suspension was homogenized. The resulting samples were centrifuged at 10,000× *g* for 20 min. All operations took place at 4 °C. The protein concentration in tissue lysates was 2 mg/mL.

Mitochondrial lysates were prepared after isolation of rat heart mitochondria from each experimental group. Heart mitochondria were isolated from Wistar rats (200–250 g, 1.5–2 months of age) by differential centrifugation, which consisted of three stages. The isolation medium contained 75 mM sucrose, 10 mM Tris-HCl (pH 7.4), 225 mM mannitol, 0.5 mM EDTA, 0.5 mM EGTA, and 0.1% BSA. The deposition of nuclei, damaged mitochondria, and blood took place at 1000× *g* for 10 min. Then, the mitochondria were sedimented at 8500× *g* for 10 min. The sedimented mitochondria were washed with an isolation medium without EGTA, EDTA, and BSA. All operations took place at 4 °C. The aliquots of isolated intact RHM (2 mg/mL) from each group were transferred in Eppendorf tubes and solubilized in Laemmli buffer (Bio-Rad, Hercules, CA, USA).

The samples of mitochondrial protein (20 μg) were applied to each line and subjected to electrophoresis and then to Western blot analysis. The polyclonal anti-ANT2, anti-VDAC2, and PHB antibodies were from Abcam (Cambridge, UK). The monoclonal anti-ATP5F1 (subunit *b* Fo sector of ATPase), ATP5G (subunit *c*), and anti-ATPB (subunit *β*) antibodies were from Abcam (Cambridge, UK). The polyclonal Tom20 (Santa-Cruz, Dallas, TX, USA) and monoclonal GAPDH (Cell Signaling, Danvers, MA, USA) antibodies were used as a loading control.

### 2.5. BNE

Blue Native Electrophoresis (BNE) was performed as described [3]. An HMW Calibration Kit for Native Electrophoresis (Sigma-Aldrich, St. Louis, MO, USA) was used as molecular markers. The gel buffer containing 150 mM Bis-Tris pH 7.0 and 10% digitonin (Sigma, Saint-Louis, MO, USA) were added to the mitochondrial suspension. After 10 min of incubation at a temperature of 4 °C, the samples were centrifuged at 10,000× *g* for 10 min. Samples were applied to 3–13% gradient gel, with 100 μg of the sample per lane. The activity of the Complexes I and V was determined by staining the gels with the appropriate substrates. The gel was stained for ~10–30 min for the detection of activity of CI by buffer containing 100 mM Tris-HCl, pH 7.4, 0.14 mM NADH, 1 mg/mL NBT (nitro blue tetrazolium chloride). For the detection of CV activity, the gel was stained for 16 h with a buffer containing 10 mM ATP, 35 mM Tris (HCL), 270 mM glycine, 14 mM MgSO4, and 0.2% Pb(NO3)_2_.

### 2.6. Liquid Chromatography and Mass Spectrometry

The samples were separated from proteolytic fragments by liquid chromatography (C18 column, 5 μm; pore size 100 Å; Phe-nomenex, USA) and analyzed by mass spectrometry (LCMS). The hydrolysate was fractionated on an Acclaim PepMap RSLC (50 μm × 15 cm) C18 column (2 μm; pore size, 100 Å; Thermo Scientific, USA) equipped with an Acclaim PepMap 100 (75 μm × 2 cm) C18 concentrating precolumn (3 μm; pore size, 100 Å) using an Easy Flow Nanotube nLC 1000 system (Thermo Scientific). The optained peptides were analyzed using mass spectrometer with the orbital trap (Orbitrap Elite; Thermo Scientific, Germany). Panoramic spectra were recorded in the *m*/*z* range of 300 to 2000 with a resolution of 240,000. An HCD camera was used to perform ion fragmentation. A resolution of 60,000 was adopted to record the fragmentation spectra. The data were processed using the Xcalibur (Thermo Scientific) and PEAKS Studio 7.5 (Bioinformatics Solution Inc., Canada) programs. In accordance with the search parameters for the identification of the peptide, PEAKS Studio 7.5 software was used [32].

### 2.7. Statistical Analysis

For statistical analysis, relative levels of protein phosphorylation were expressed as mean ±SD from of 3 independent experiments with samples being run in duplicate. For statistical analysis, we used one-way ANOVA and a proper post-hoc analysis (Student–Newman–Keuls).

## 3. Results

In this work, heart failure was achieved by the injection of isoproterenol (ISO), a model accepted in the world scientific community [23,30]. In order to show that heart failure was achieved in our experiments, we investigated the effect of AX on the change in the level of proteins in tissue lysates of the heart from each group of rats during ISO-induced oxidative stress.

**Figure 1:**
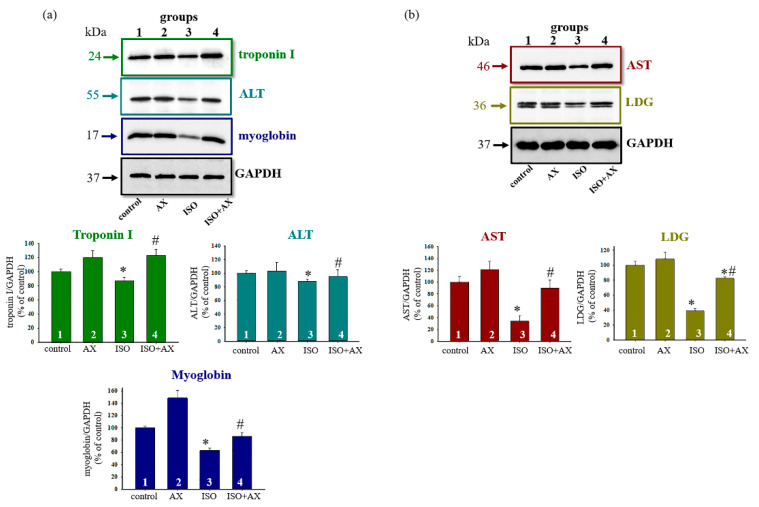
Effects of the administration of AX and injection of ISO on the content of Troponin I, ALT, Myoglobin, AST, and LDG in rat heart tissue. Protein samples were extracted and subjected to Western blotting. The immunodetection of GAPDH was used as a loading control. (**a**)—Immunostaining with Troponin I, ALT, Myoglobin antibodies and GAPDH (upper part); quantification of immunostaining by computer-assisted densitometry presents as a ratio of proteins to GAPDH (low part), (**b**)—Immunostaining with AST and LDG antibodies and GAPDH (upper part); quantification of immunostaining by computer-assisted densitometry presented as a ratio of proteins to GAPDH (low part). Bar graphs represent the levels of appropriate proteins; the data are presented as the means ± SD of three independent experiments. * *p* < 0.05 significant difference in the protein level in comparison with the control (group 1). # *p* < 0.05 compared to RHM isolated from ISO-injected rats (group 3). The statistical significance of the differences between the pairs of mean values was evaluated using the Student-Newman-Keul test.

For this, antibodies to alanine aminotransferase (ALT), aspartate aminotransferase (AST), lactate dehydrogenase (LDG), troponin I, and myoglobin were used (Figure 1). Figure 1a,b demonstrates a Western blot stained with troponin I, ALT, myoglobin, AST, and LDG antibodies (upper part). GAPDH was used as a loading control. Figure 1 (low part) show the immunostaining results obtained by computed densitometry and are presented as the ratio of proteins (optic density) to GAPDH. We observed that the content of each protein decreased in the presence of ISO compared to control (columns 3 vs. 1), while AX in combination with ISO increased the level of proteins in comparison with ISO alone (columns 4 vs. 3). AX abolished ISO-induced oxidative stress in the tissue lysates of the heart from each group of rats.

**Figure 2:**
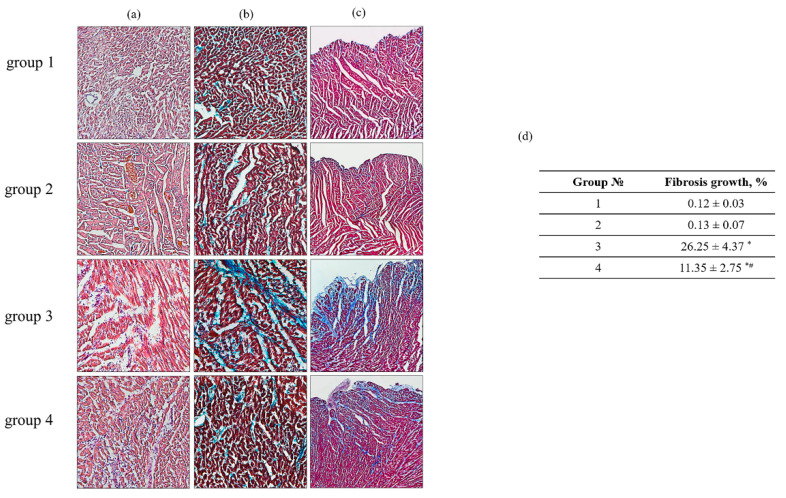
Histological samples of heart ventricles in rats of the control and experimental groups. (**a**)—H&E (cell nuclei are in blue, erythrocytes are in red, muscle tissue is in pink); (**b**)—Masson’s trichrome staining (collagen/fibrosis is stained with blue, muscle and other tissues are stained with red, and cell nuclei are stained with brown); (**c**)—Lillie’s trichrome staining (collagen/fibrosis is shown in blue; muscle and other tissues are in red-brown; cell nuclei are in brown-black); (**d**)—a table reflecting fibrosis growth in the tissue of the left ventricle from each group of rats. Data are presented as means ± SDs of five independent experiments. The numbers on the graphs represent the percentage of fibrous growth in the tissues of each group. * *p* < 0.05 indicates a significant difference in the tissue damage relative to the control (group 1). # *p* < 0.05 compared to RHM isolated from ISO-injected rats (group 3). The statistical significance of the differences between the pairs of the mean values was evaluated using an ANOVA type 2 (Student–Newman–Keuls) test.

To assess the degree and nature of myocardial damage as well as to identify the localization of the damage, transmural histotopograms of left ventricular samples of all groups were analyzed and subepicardial, median, and subendocardial myocardial zones were compared (Figure 2). To identify the degree of fibrosis and myocardial hypertrophy, the zones with the transverse or longitudinal section of the muscle fibers of the heart were investigated. The obtained results showed that all histological parameters of the studied groups corresponded to the standards of the structure and architectonics of the myocardium of this age group of rats. The comparative analysis of samples from groups 1 and 2 of rats did not reveal any differences in all studied myocardial zones. However, in the samples of rat LV from groups 3 and 4, subendocardial myocardial damage was observed. It should be noted that in the LV sample of rat from group 4, fibrotic myocardial damage was less pronounced compared with LV from group 3 (LV tissue from group 4 vs. group 3).

In addition, in the LV samples of rats from group 4, the subepicardial and midline zones of the heart wall were not damaged in comparison with the LV samples of group 3, in which signs of fibrotic changes in the middle region of the myocardium and the fusion of edematous muscle fibers with the appearance of subsegmental contractures were observed. The degree of increase in fibrotic changes in the LV samples of rats from group 4 decreased by 43% related to the LV samples of rat from group 3 (Figure 2d). Analyzing the histotopograms of the samples, we found that signs of myocardial hypertrophy were observed in rats in both group 3 and group 4. However, in LV samples of rats from group 4, this process was less pronounced and limited to the subenocardial and median zone compared to the samples of the LV in rats of the group 3, where the damages were total. AX abolished ISO effect and decreased the damage of heart.

**Figure 3:**
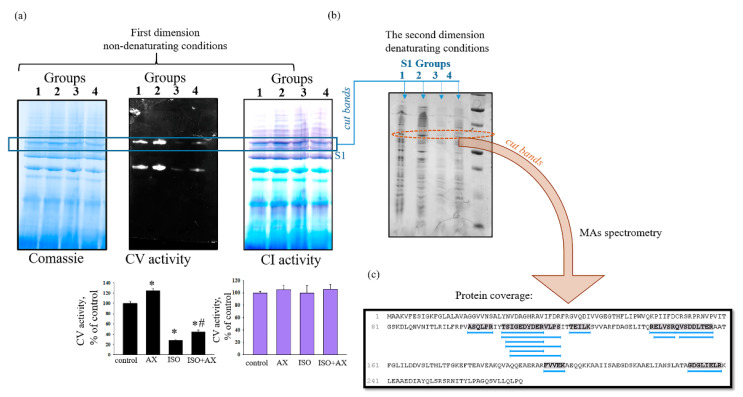
The effect of chronic treatment of AX on the activity of supercomplexes (SCs) in rat heart mitochondria in heart failure. (**a**)—the first dimension after separation of the mitochondrial suspension by BNE (non-denaturing condition) Coomassie staining, Complex V and I activity (upper part); diagram reflecting the change in the activity of Complexes V and I (low part). Optic density of Coomassie bands was used as a loading control; (**b**)—the second dimension (Laemmli system under denaturing conditions) after cutting strips from the gel after the first measurement; (**c**)—the analysis of the amino acid sequence (protein coverage). Data are presented as means ±SDs of four independent experiments. * *p* < 0.05 indicates a significant difference in the protein level relative to the control (group 1). # *p* < 0.05 compared to RHM isolated from ISO-injected rats (group 3). The statistical significance of the differences between the pairs of the mean values was evaluated using an ANOVA type 2 (Student–Newman–Keuls) test.

Further, we checked the effect of chronic treatment of AX on the activity of supercomplexes (SCs) in rat heart mitochondria in heart failure (Figure 3). The samples of native heart mitochondria isolated from all experimental groups of rats were separated by Blue Native Electrophoresis (under non-denaturating conditions) into SCs as described in the Section 2. At first, we measured the activity of the Complexes I and V of the respiratory chain in the gels (Figure 3a, upper part) in the separated SCs of RHM. Figure 3a low part demonstrates the diagrams containing the alteration of Complexes I and V activities in the RHM isolated from each group rats. Coomassie bands were used as a loading control. We noticed that the activity of Complex V decreased by 70% in mitochondria isolated from rats of group 3 in comparison with the control (RHM from group 1). In the RHM of the group 4 rats (ISO + AX), the activity of Complex V increased by 40% in comparison with RHM of rats from group 3 (ISO injection). However, the activity of the Complex I in SCs did not change in RHM isolated from each group.

Next, we cut out bands from gel (the first dimension) according to changes in the activity of Complex V and performed separations in the Laemmli system under denaturing conditions (Figure 3b, second dimension). Then, the obtained gels were stained with Coomassie Brilliant Blue R-250 staining solution, where the changed bands by staining were cut out and mass spectrometric analysis was performed. Figure 3c shows the analysis of the amino acid sequence (protein coverage).

The analyzed results indicating protein candidates in the test samples are shown in Table 1 and Table 2. Table 1 demonstrates that the protein was identified as prohibitin (PHB) with molecular weight 29,820 Da (~30 kDa). However, other proteins were observed in the sample, such as ADP/ATP translocase 2, ATP synthase subunit beta, ATP synthase subunit alpha, NADH dehydrogenase (Ubiquinone) Fe-S protein 3, and voltage-dependent anion-selective channel protein 1 (VDAC1) (Table 2). Some proteins are either Complex I or V proteins.

**Figure 4:**
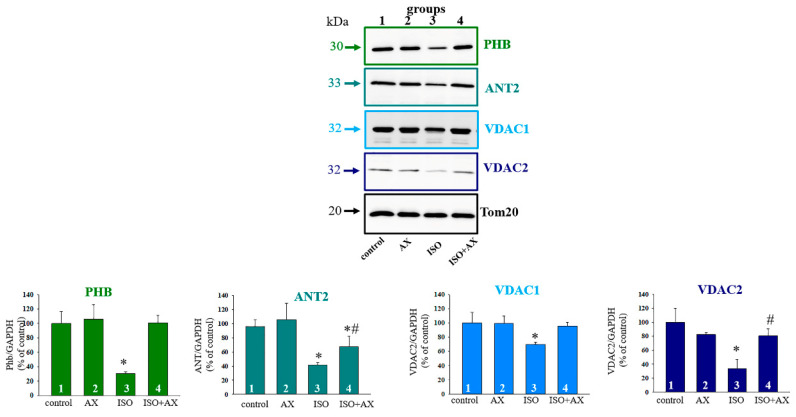
Effects of the administration of AX on the content of PHB, ANT2, VDAC1, and VDAC2 in rat heart mitochondria in heart failure. Upper part—immunostaining with PHB, ANT2, VDAC1, VDAC2, and Tom20 antibodies; Low part—quantification of immunostaining by computer-assisted densitometry presented as a ratio of proteins to Tom20. Bar graphs represent the levels of appropriate proteins; the data are presented as the means ±SD of three independent experiments. * *p* < 0.05 significant difference in the protein level in comparison with the control (group 1). # *p* < 0.05 compared to RHM isolated from ISO-injected rats (group 3). The statistical significance of the differences between the pairs of mean values was evaluated using the Student-Newman-Keul test.

It is known that there are PHB partners, such as ANT2, VDAC2 [33], and others. PHB is also found in the mitochondrial inner membrane and forms a complex with VDAC2 and ANT2, which are involved in apoptosis [34]. Moreover, PHB regulates of the activity of OXPHOS by interacting with some subunits of the OXPHOS complexes [9]. Therefore, we investigated the changes in these proteins and PHB as well as some subunits of Complex V under our experimental conditions. Figure 4 (upper part) shows Western blots of PHB, ANT2, VDAC1, and VDAC2 in RHM isolated from each group of rats. A quantitative analysis of the level of proteins is shown in Figure 4 (low part). Protein bands were quantified after normalization with respect to Tom20. As seen from Figure 4, the levels of PHB, ANT2, VDAC1, and VDAC2 decreased by 70%, 60%, 40%, and 60%, respectively, in RHM isolated from the ISO-injected rats compared to the control group of rats (RHM from group 3 vs. group 1). The administration of AX increased the level of these proteins by three times, 40%, 30, and two times, respectively, relative to the effect of ISO alone (RHM from group 4 vs. group 3).

**Figure 5:**
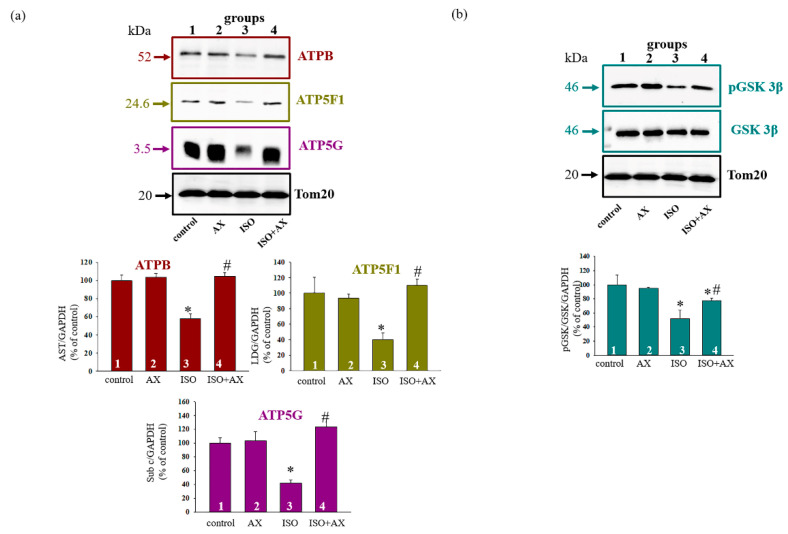
Effects of the administration of AX on the content of ATPB, ATP5F1, ATP5G, pGSK3β and GSK3β in rat heart mitochondria in heart failure. (**a**)—immunostaining with ATPB, ATP5F1, ATP5G, and Tom20 antibodies (upper part); low part—quantification of immunostaining by computer-assisted densitometry presented as a ratio of proteins to Tom20; (**b**)—immunostaining with pGSK3β, GSK3β, and Tom20 antibodies (upper part); low part—quantification of immunostaining by computer-assisted densitometry presented as a ratio of proteins to Tom20. Bar graphs represent the levels of appropriate proteins; the data are presented as the means ± SD of three independent experiments. * *p* < 0.05 significant difference in the protein level in comparison with the control (group 1). # *p* < 0.05 compared to RHM isolated from ISO-injected rats (group 3). The statistical significance of the differences between the pairs of mean values was evaluated using the Student-Newman-Keul test.

Figure 5a demonstrates the change in the level of subunits β, *b*, and *c.* The upper part represents Western blot of subunits while the lower part shows a quantitative analysis of the proteins normalized to Tom20. We noticed that the level of subunits β, *b*, and *c* diminished in RHM from the ISO-injected rats by 40%, 60%, and 60%, respectively, relative to the control (RHM from group 3 vs. group 1). The combined effect of AX and ISO increased the level of subunit β by 50%, but subunits *b* and *c* by three times in RHM compared to RHM from the ISO-injected rats (RHM from group 4 vs. group 3). AX abolished the effect of ISO and increased the content of proteins in RHM.

Many functions of PHBs have been proposed, including a role in the regulation of the cell cycle [35,36] and the transduction of a transmembrane signal [37,38]. Here, we examined how the level of phosphorylated GSK3β changed in the presence of AX (Figure 5b). Figure 5 (upper part) shows Western blots stained with antibodies to pGSK3β and GSK3β. Tom20 was used as a loading control. A quantitative analysis of the ratio of pGSK3β to GSK3β is presented in the lower parts of Figure 5, showing that the ratio of pGSK3β/GSK3β decreased by ~50% in the RHM after ISO injection compared to the control (RHM from group 3 vs. group 1), whereas AX increased this ratio by 50% in RHM isolated from ISO-injected rats relative to ISO alone (RHM from group 4 vs. group 3). The administration of AX eliminated the harmful effect of ISO in RHM.

## 4. Discussion

Heart failure is one of the leading causes of morbidity and mortality worldwide and is considered an urgent problem not only among the elderly, but also among young people. Various types of damage to the heart, such as arterial hypertension, coronary artery disease, and cardiomyopathy, among others, lead to heart failure [39,40]. It is especially important to monitor the health of the heart in cancer patients. In this regard, there are strategies that can reduce mortality and the incidence of cardiovascular disease in cancer patients using antidiabetic drugs such as glyflozins [41].

It is known that mitochondria are the main organelles responsible for the production of ATP in the cell. Mitochondria are vulnerable to oxidative damage, which can lead to structural changes that become more pronounced in pathological conditions [42]. Various pathological conditions result in mitochondrial dysfunction, which leads to a decrease in ATP production and cell death. Therefore, the main task of researchers is to find the targets of action on the cell in order to improve the work of the damaged organ (for example, heart). In this work, we identified a protein with a molecular weight of 30 kDa that changed its content in heart failure. Heart failure was caused by ISO injection. The changes of proteins in tissue lysates of the LV of the heart rats (Figure 1) and histological analysis of tissue in the LV of the heart rats (Figure 2) prove that heart failure has been achieved. Mass spectrometry analysis showed that the investigated protein is PHB (Figure 3 and Table 1).

It is considered that PHBs represent a highly conserved group of proteins, ubiquitously expressed in many cell types, which are localized in the inner mitochondrial membrane, nucleus, and plasma membrane. PHBs are involved in the regulation of activity of Complexes respiratory chain by interacting with some subunits of the complexes [9]. There are two isoforms of PHB. PHB1 and PHB2 are two highly homologous subunits of the eukaryotic mitochondrial PHB complex. It has also been shown that PHB1 has a cardioprotective and anti-inflammatory effect in the heart, which is partly due to its effect in maintaining oxidative phosphorylation and controlling metabolism [43]. Dechao Wu and coauthors showed that PHB2 ablation resulted in cardiac mitochondrial dysfunction and played an essential role in cardiac fatty acid metabolism homeostasis [44]. Therefore, it can be assumed that PHBs are important for the normal functioning of the heart.

It was previously reported that PHBs are closely related to some subunits of Complexes of respiratory chain [9]. It was found that PHBs are in a complex with proteins that perform various cellular functions [12]. In our study, we found that the proteins shown in Table 2 were observed in the samples after mass spectrometry. The presence of NADH dehydrogenase (Ubiquinone), ATP synthase subunit beta, and ATP synthase subunit alpha suggests that the supercomplex included Complex I and V (Table 2). Moreover, it was known that PHB co-localized with ANT2 and VDAC2 [31]. We observed that the samples after mass spectrometry contained ADP/ATP translocase 2 and Voltage-dependent anion-selective channel protein 1 (Table 2). Here, we checked how PHB, ANT2, VDAC1, VDAC2, and subunits β, *b*, and *c* changed under our experimental conditions (Figure 4 and Figure 5). In all cases, we have observed that ISO decreased protein levels, while AX restored protein levels to near control level. Whether this recovery occurs due to PHB needs to be clarified in our further studies. However, it can be assumed that PHB is important for the normal functioning of the heart and may be a target of the action of AX.

Recently, it was reported that PHB took part in the regulation of signal transduction [37,38]. Moreover, it is known that there are proteins localized in mitochondria are subject to phosphorylation, which ultimately can determine cell viability [45]. PHBs can be classified as such proteins. PHB enhances signaling pathways, implicating cellular apoptosis, survival, and differentiation [46,47,48]. In addition, many processes in mitochondria that occur in various pathologies are regulated by phosphorylation/dephosphorylation of glycogen synthase kinase 3 (GSK3β) [49]. Several membrane-bound proteins are phosphorylated by GSK3β, e.g., VDAC [50,51] and ANT [52]. Since GSK3β is able to phosphorylate ANT and VDAC, and PHB forms a strong complex with these proteins, GSK3β can participate in PHB phosphorylation. However, this hypothesis needs to be checked in further research. We have shown that the ISO injection resulted in dephosphorylation and, consequently, the activation of GSK3β, while the combination AX with ISO led to GSK3β phosphorylation, and consequently, its inactivation. We hypothesize that cardioprotection in RHM can be regulated by different kinases, in particular GSK3β, which, in turn, will be able to activate or inactivate mitochondrial proteins by phosphorylation/dephosphorylation.

## 5. Conclusions

In this study, we identified a protein that changed its content in heart failure. This protein was PHB that can be localized in the mitochondria, nucleus, and plasma membrane and perform many functions in cell. PHB forms a complex with proteins of the outer and inner mitochondrial membranes and with some subunits of the complexes of respiratory chain. ISO caused a decrease in the level of PHB and the protein-partners, while AX restored their content almost to the level of control in the mitochondria of ISO-injected rats. We hypothesized that PHB could be a target for AX in heart failure. This hypothesis requires further investigation. Taken together, the data presented here indicate a great interest in the study of PHB in mitochondria.

## Figures and Tables

**Table 1:** Identification of 30 kDa protein in RHM by mass spectrometry as Prohibitin (PHB).

Protein ID	Accession	Score (%)	10lgP	Coverage (%)	Peptides	Unique	PTM	Avg. Mass	Description
43163	P67779|PHB_RAT	98.9	109.67	10	14	10	N	29,820	Prohibitin OS = Rattus norvegicus OX = 10116 GN = Phb PE = 1 SV

**Table 2:** The analysis results indicating protein candidates in the mitochondrial samples.

Protein ID	Accession	Score (%)	10lgP	Coverage (%)	Peptides	Unique	PTM	Avg. Mass	Description
43143	Q09073|ADT_RAT	98.7	98.38	12	14	13	Y	32,901	ADP/ATP translocase 2 OS = Rattus norvegicus OX = 10116 GN = Slc25a5 PE = 1 SV = 3
43089	P10719|ATPB_RAT	99.1	215.32	23	37	37	Y	56,354	ATP synthase subunit beta, mitochondrial OS = Rattus norvegicus OX = 10116 GN = Atp5f1b PE = 1 SV = 2
43087	tr|F1LP05|F1LP05_RAT	99.1	151.88	26	26	24	Y	59,813	ATP synthase subunit alpha OS = Rattus norvegicus OX = 10116 GN = Atp5f1a PE = 1 SV = 1
43230	tr|D3ZG43|D3ZG43_RAT	99.1	146.89	33	28	27	N	30,226	NADH dehydrogenase (Ubiquinone) Fe-S protein 3 (Predicted), isoform CRA_c OS = Rattus norvegicus OX = 10116 GN = Ndufs3 PE = 1 SV = 1
43093	P35435|ATPG_RAT	98.7	96.07	14	9	9	Y	30,191	ATP synthase subunit gamma, mitochondrial OS = Rattus norvegicus OX = 10116 GN = Atp5f1c PE = 1 SV = 2

## Data Availability

The data presented in this study are contained within this article.
